# Impact of the COVID-19 Pandemic on Acute Ischemic Stroke Presentation, Treatment, and Outcomes

**DOI:** 10.1155/2021/8653396

**Published:** 2021-07-29

**Authors:** Timothy G. White, Gabriela Martinez, Jason Wang, Michele Gribko, Artem Boltyenkov, Rohan Arora, Jeffrey M. Katz, Henry H. Woo, Pina C. Sanelli

**Affiliations:** ^1^Department of Neurosurgery, Donald and Barbara Zucker School of Medicine at Hofstra/Northwell, USA; ^2^Center of Health Innovations and Outcomes Research, Feinstein Institutes for Medical Research, Manhasset, NY, USA; ^3^Siemens Healthineers, Malvern, PA, USA; ^4^Department of Radiology, Donald and Barbara Zucker School of Medicine at Hofstra/Northwell, USA; ^5^Department of Neurology, Donald and Barbara Zucker School of Medicine at Hofstra/Northwell, USA

## Abstract

**Introduction:**

The World Health Organization declared COVID-19 a global pandemic last year. While a clear impact of COVID-19 on the declining stroke volume has been reported, its overall impact on stroke presentation and clinical outcomes has not been established. The purpose of this study was to assess the impact of COVID-19 on acute ischemic stroke volume, presentation, treatment, and outcomes at comprehensive stroke centers.

**Methods:**

A retrospective review of patients with a discharge diagnosis of acute ischemic stroke from the Get With The Guidelines database was performed from January 1, 2019, to July 1, 2020. The following time periods were defined: Pre-COVID (January/February), Peak-COVID (March/April), and Post-COVID (May/June). Bivariate analyses were performed comparing the 2020 and 2019 time periods to determine differences in stroke volume, presentation, treatment, and outcomes.

**Results:**

Stroke volumes were significantly lower during the Peak-COVID period in 2020 compared to that in 2019, with an absolute decline of 49.5% (*P* < 0.001). Patients were more likely to present after 24 hours from last known well during the 2020 Peak-COVID period (*P* = 0.03). However, there was not a significant difference in the rate of treatment with either the tissue plasminogen activator (tPA) or mechanical thrombectomy during the Peak-COVID period. Interestingly, relative treatment rates increased during the 2020 Post-COVID period to 11.4% (*P* = 0.01).

**Conclusions:**

The overall ischemic stroke volume decreased during the pandemic, and patients had a tendency to present later, beyond eligible treatment windows. However, rates of treatment, patient demographics, and stroke outcomes did not significantly change when compared to the prior year.

## 1. Introduction

In early March 2020, the World Health Organization (WHO) declared the coronavirus disease of 2019 (COVID-19) a global pandemic. Since then, hospitals and surgical departments around the world were forced to restructure the way that healthcare is delivered. This was particularly true when it came to the delivery of emergency care for stroke and cerebrovascular diseases. Numerous organizations including the American Heart Association/American Stroke Association (AHA/ASA) disseminated guidelines on procedural safety optimization while simultaneously, reports suggested an increased risk of thromboembolic complications due to the disease and COVID-19-related stroke data being increased [1, 2]. However, a decreased incidence of overall stroke presentations was observed and many institutions experienced a sharp decline in stroke volume during the pandemic [3–11]. Due to the fear of the pandemic and overwhelmed healthcare systems, patients with stroke and cerebrovascular disease may have been extremely hesitant to seek emergency hospital care, especially if it were not a life-threatening symptom [12].

Several publications in the literature have reported a decrease in the presentation of patients with acute ischemic stroke and a decrease in the number of stroke interventions. In particular, a large French registry demonstrated a decreased number of ischemic stroke patients along with a decreased number of mechanical thrombectomies, increased time from imaging to groin puncture, and slower speed of transfer [13]. Similar data has emerged from multiple other regions [3, 4]. One large international registry of all ischemic stroke patients being treated with endovascular therapy (EVT) suggested that patients presenting in areas with high COVID-19 volume were less likely to get intravenous the tissue plasminogen activator (IV-tPA) but were otherwise similar to patients presenting in the low-COVID-19 regions [6]. This series, however, only included patients undergoing EVT and ultimately compared patients from different regions, lacking the internal control of comparing the same institution over temporal periods [6].

Significant concerns have been raised that delayed emergency care may negatively affect the stroke severity at presentation with the worse National Institute of Health Stroke Scale (NIHSS) and extended symptom onset times that could disqualify patients from treatment with IV-tPA and/or EVT, thus leading to overall worse outcomes of stroke patients. While a clear impact of COVID-19 on declining stroke volume exists, its overall impact on the stroke presentation characteristics and, importantly, clinical outcomes has not been established. Furthermore, the period after the initial COVID-19 surge in the post-COVID-19 months has not been described. The purpose of this study was to assess the impact of the COVID-19 pandemic on acute ischemic stroke volume, presentation, treatment, and outcomes at comprehensive stroke centers during the 2020 Pre-, Peak-, and Post-COVID time periods, compared to the prior year (2019).

## 2. Materials and Methods

A retrospective review of consecutive patients in the Get With the Guidelines (GWTG) stroke database from two comprehensive stroke centers in the New York City metropolitan area was performed from January 1, 2019, to July 1, 2020. Inclusion criteria were adult patients (≥18 years old) with the discharge diagnosis of acute ischemic stroke. Exclusion criteria were discharge diagnosis of hemorrhagic stroke or transient ischemic attack or patients with in-hospital stroke onset. Institutional review board approval was obtained with waiver of consent given at the retrospective data collection.

Data was extracted from the GWTG stroke database and included stroke volume by month, patient age and sex, stroke severity (NIHSS), time from last known well (LKWA) to hospital arrival, mode of patient arrival (emergency medical services, transfer, private, or unknown), treatment type (IV-tPA, EVT, and none), and outcomes (discharge disposition and length of stay).

The first positive-tested COVID-19 case in the New York City region was documented on March 1, 2020, which defined the starting point of the Peak-COVID period. The Pre-COVID period in year 2020 was defined prior to the first positive-tested COVID-19 case as January 1^st^–February 29^th^. The Post-COVID period was defined as the decline after the first wave of the pandemic from May 1^st^ to June 30^th^ when the daily new COVID case volume started to drop [14]. Therefore, the dataset was split into 3 specified time periods as Pre-COVID (January–February), Peak-COVID (March–April), and Post-COVID (May–June).

### 2.1. Statistical Analysis

In the bivariate analyses, all variables were analyzed as categorical variables. Age was grouped as 18–39, 40–59, 60–79, and 80+. NIHSS was assigned as a categorical variable as either 0–9, 10+, or unknown. LKWA was categorized into 5 groups (measured in hours): 0–4.5, 4.5–6, 6–24, >24, or unknown. Length of stay (LOS) was dichotomized as 0–7 or 8+ days, and discharge disposition was defined as home, expired, or other (rehabilitation or long-term care facility).

Bivariate analyses were performed comparing the 2020 time periods (Pre-COVID, Peak-COVID, and Post-COVID) with the 2019 time periods to account for monthly and seasonal variation. Additional analyses were performed in 2020 comparing the Pre-COVID to the Peak-COVID and Post-COVID time periods to account for possible yearly variation. Furthermore, mean LOS was analyzed and compared between 2020 and 2019 time periods as a focused comparison between the 2020 time periods. Data analyses were performed using Student's *t*-test for continuous variables, chi-square tests for categorical variables, and Fischer's exact tests when cell numbers were less than 5.

Logistic regression models with stepwise selection were used to determine the patient factors associated with in-hospital mortality during the following time periods: (1) 2020 Peak-COVID time period and (2) control time period (January 1–June 30, 2019). Age (0–70, >70), and sex variables were treated as dichotomized variables for the purposes of the regression analysis. A *P* value of <0.05 was considered statistically significant.

## 3. Results

Overall, there were a total of 440 stroke patients who presented in 2020 from January 1^st^–June 30^th^ compared to 596 stroke patients during the same months in 2019.  Table 1 demonstrates the stroke volume in the Pre-COVID, Peak-COVID, and Post-COVID time periods in 2020 and 2019. Stroke volume was statistically different over the various time periods, with the greatest percent difference in stroke volume occurring during the 2020 Peak-COVID time period, compared to the same time period in 2019 (*P* < 0.001). There were 100 patients who presented with acute ischemic stroke during the 2020 Peak-COVID time period compared to 198 stroke patients in 2019, representing almost half (50.5%) of the stroke volume.

### 3.1. Patient Characteristics and Stroke Severity

Table 2 compares patient characteristics (age, sex, mode of arrival, and LKWA) and stroke severity (NIHSS) in 2020 and 2019 Pre-COVID, Peak-COVID, and Post-COVID time periods. Overall, there was no statistically significant differences in age, sex, NIHSS, and mode of arrival. However, statistical significance was observed with LKWA during the Pre-COVID (*P* = 0.042) and Peak-COVID (*P* = 0.03) time periods. In particular, a higher proportion of patients presented in the late time window > 24 hours after symptom onset, while a smaller percentage of patients presented during the eligible treatment windows for IV-tPA (0–4.5 hours) and EVT (0–24 hours). During the 2020 Post-COVID period, there were no significant differences in the LKWA, with similar proportions of patients presenting with LKWA 0–24 hours, compared to 2019.

The mode of patient arrival was similar during the 2020 Peak-COVID time period compared to the same time period in 2019. There was a trend towards fewer patients being transferred (22% in 2020 versus 34% in 2019), though it did not reach statistical significance. During the Post-COVID time period, there was a trend towards more patients arriving via EMS (35.5% in 2020 versus 22.7% in 2019) with relatively fewer patient transfers; however, this was not significant (*P* = 0.09).

### 3.2. Stroke Treatment and Outcomes

Although the proportion of patients treated with IV-tPA did not significantly differ between Pre-COVID, Peak-COVID, and Post-COVID time periods in 2020 and 2019 (Table 2), there was a trend towards fewer EVT performed during the 2020 Peak-COVID time period (2%) compared to the same time period in 2019 (7%) (*P* = 0.07). There was also a statistically significant increase in patients undergoing EVT during the 2020 Post-COVID time period (11.4%) compared to the same time period in 2019 (3.9%) (*P* = 0.01). Although the NIHSS and LKWA were similar in 2019 and 2020 Post-COVID time periods (May–June of respective years), relatively more patients were treated with EVT in 2020 (11.3%) than in 2019 (3.9%).

Stroke outcomes were assessed by discharge disposition and LOS. There was no significant difference between the proportion of patients discharged home in 2020 Pre-COVID, Peak-COVID, and Post-COVID time periods compared to those in 2019. Overall, mortality was similar particularly during the Peak-COVID period (12% in 2020 versus 10% in 2019). LOS was similar in 2020 and 2019 Pre-COVID, peak-COVID, and Post-COVID time periods. The average LOS during the 2020 Peak-COVID time period was 7.4 days compared to 9.7 days in 2019, representing borderline statistical significance (*P* = 0.05).

### 3.3. Time Period Comparisons in 2020

Within 2020, comparing the Pre-COVID, Peak-COVID, and Post-COVID time periods revealed similar age, sex, and stroke severity as seen in Table 3. Interestingly, in the Post-COVID time period, patients were more likely to undergo EVT (11.4%) compared to 2% and 8% during the Peak- and Pre-COVID time periods, respectively (*P* = 0.03). There was no significant difference in the mode of arrival as well as discharge disposition. Mean LOS was statistically longer during the Pre-COVID time period than during the Peak-COVID and Post-COVID time periods, with 48.7% of patients staying for longer than seven days in the Pre-COVID time period compared to 35% and 31.9% during the Peak-COVID and Post-COVID time periods, respectively (*P* = 0.004). Mean LOS in the Pre-COVID time period was 10.37 days compared to 7.20 days during the Peak-COVID and Post-COVID time periods averaged (*P* = 0.0002).

### 3.4. In-Hospital Stroke Mortality Analyses

We developed two separate stepwise logistic regression models for in-hospital mortality for the control (January–June 2019) and the 2020 Peak-COVID time periods. As shown in Table 4, for the control time period, age (≥70 years), NIHSS (≥10), LOS (≥7 days), and LKW (≥24 hours) were significantly associated with in-hospital mortality, while only age and LOS were significantly associated with in-hospital mortality during the 2020 Peak-COVID time period, possibly due to the smaller sample size.

## 4. Discussion

The impact of COVID-19 on declining stroke volume has been reported by multiple studies in the literature. However, some publications have emerged regarding the association of COVID-19 increasing the risk of thromboembolic disease, particularly acute ischemic stroke [2, 15]. Early studies from Barcelona, Spain, and Hong Kong, China, were the first to report a decrease in the number of stroke codes and that patients were presenting to the hospital later after the onset of their symptoms [9, 16]. This was echoed by follow-up studies such as a cross-sectional study in France that demonstrated a 21% decrease in mechanical thrombectomy volume during COVID-19 with overall delays in treatment times as well [13]. Other studies even demonstrated a decrease in 911 calls for patients with strokes and a decrease in the number of patients presenting with transient ischemic attack [16]. Overall, it seems that the entire stroke system of care has been impacted by the COVID-19 pandemic with decreases in emergency room presentations, ischemic stroke admissions, mechanical thrombectomies, stroke codes, and even 911 activations all being demonstrated [6, 9, 10, 17]. However, few studies to date have discussed outcome variables for patients presenting with acute ischemic stroke (AIS) during the peak of the COVID-19 pandemic or the changing stroke characteristics during the initial COVID-19 surge recovery period.

Studies have suggested that the decline in stroke volume during the COVID-19 pandemic and the complexity behind the management of these patients may lead to a temporal impact on the presentation and treatment in patients with AIS [6, 9]. One main concern is that patients were potentially presenting outside of key treatment windows for IV-tPA and EVT during the COVID-19 pandemic [18]. In this study, we demonstrated similar findings to the previously mentioned literature with patients being more likely to present after 24 hours from last known well and that the total stroke volume was lower at the peak of the COVID-19 pandemic in March and April of 2020 compared to 2019. Despite a trend to later presentation with patients being more likely to present after 24 hours, there was no significant differences in treatment, likely due to the small sample size.

Otherwise, stroke demographics including severity of stroke at presentation measured by NIHSS were relatively similar among the different time periods considered in the analyses. Interestingly, there was a trend towards a decreased number of transfers during the Peak- and Post-COVID periods compared to the Pre-COVID period in 2020 as well as in 2019. The potential decrease in transfer volume is likely due to the practice in the initial pandemic period to limit interfacility transfers to mitigate the risk of infection transmission. Length of stay and in-hospital mortality were used as surrogates for patient outcomes. In-hospital mortality and discharge location were not significantly different when comparing the 2020 Peak-COVID and control time periods. Furthermore, our stepwise regression analysis demonstrated that drivers of in-hospital mortality were similar in the 2020 Peak-COVID and control time periods. Length of stay trended toward being lower during the Peak-COVID and Post-COVID periods. This may have been due to a healthcare-wide initiative to discharge patients in a timely manner to make beds available for incoming patients due to overcapacity limits.

As far as we are aware, this study is the first study to address the Post-COVID time period after the initial surge of COVID-19 in the New York metropolitan area. Interestingly, we did find that the rate of stroke patients treated with EVT increased during the Post-COVID time period when compared to the same months in 2019 and earlier in the 2020 Pre-COVID period. The rate of EVT treatment was significantly higher in the 2020 Post-COVID time period compared to either 2019 or 2020 earlier periods (11.4% compared to 2%–7% other time periods in 2020). This is remarkable in light of numerous reports describing the presence of “COVID strokes” where young individuals were presenting with atypical thromboembolic events [15, 19]. Importantly, it may be possible that even patients with mild or asymptomatic COVID-19 have an altered coagulation profile or continue to have aberrant coagulation even after disease recovery [2]. While we cannot prove this association to be true, it does raise significant concerns and is consistent with previously reported anecdotal evidence [19]. However, patients were more likely to present within 24 hours of symptom onset during the Post-COVID period in 2020; this may have subsequently qualified them for thrombectomy in a greater number than patients presenting in Peak-COVID 2020.

Clearly, COVID-19 has demonstrated a multifactorial impact on the stroke chain of care and acute ischemic stroke workflows. It is likely that the numerous reports of COVID-19-associated stroke and increase in thromboembolic disease make up a small proportion of overall stroke patient volume [20]. While this and other studies have clearly demonstrated a downtrend in total stroke volume as well as reports of COVID-19-associated strokes, there seems to be no clear changing demographics or presenting characteristics of these patients [3, 5, 7, 8, 13, 17, 21]. Our study also suggests that COVID-19-driven strokes seem to make up a small proportion of overall stroke volume to the point where in our patient population, there was no significant change in stroke demographics. Furthermore, the findings presented herein are consistent with previous studies with a higher proportion of patients presenting outside of the 24-hour time window [13]. Results of the DAWN trial demonstrated the clear safety and efficacy of mechanical thrombectomy up to 24 hours after symptom onset for patients with AIS and large vessel occlusion; therefore, presenting outside of the 24-hour time window may prohibit patients from a lifesaving intervention [22]. Delays in stroke care have a potentially devastating impact on patient outcomes, and therefore, it is important to establish the impact of COVID-19 in stroke care to best address any challenges that may arise due to the pandemic as well as future pandemics or challenges to healthcare systems. Finally, the findings of an increase in EVT volume during the 2020 Post-COVID period, after the first wave of the pandemic, are of note and deserve further study. As prior literature has demonstrated, COVID-19 is associated with a prothrombotic state; it may need to be considered as a predisposing risk factor in patients presenting with stroke in the future. Prior studies of stroke in patients with COVID-19 have demonstrated an association between COVID-19 and in-hospital stroke rates [20].

This study has several limitations, including its retrospective nature and a limited sample size from a narrow geography of only two thrombectomy-capable stroke centers in the New York metropolitan area which limits the generalizability of these findings. The small sample size may have contributed to the differences in the logistic regression model from the 2020 Peak-COVID and control time periods. However, the findings do reflect results from high volume COVID-19 centers and, despite its small sample size, do have findings with important implications. Our study did not use patient-reported outcome information, such as the Modified Rankin Score. Nevertheless, we used discharge disposition as a surrogate of functional outcome given that several studies support discharge disposition as a good predictor of longer-term stroke outcomes [23, 24].

## 5. Conclusion

Although stroke volume decreased in 2020 during the COVID-19 pandemic, stroke patients that presented for medical care had similar characteristics of age, sex, NIHSS, and mode of arrival as in the previous year. Although LKW to arrival time was significantly different in the Peak-COVID period with a higher proportion of patients presenting >24 hours, the stroke treatment delivery rates were similar to those of the prior year. However, the percentage of treated patients increased in the 2020 Post-COVID time period. The mean LOS was significantly shorter during the Peak- and Post-COVID periods, compared to the prior year. Importantly, our analyses revealed that the main drivers of in-hospital mortality remained fairly consistent over the time periods analyzed in our study. Numerous groups have advocated for increasing public awareness regarding the association of COVID-19 to delayed stroke treatment and clinical outcomes. Thus, public education is critical, especially during this pandemic, to enhance the timely diagnosis of acute ischemic stroke so that treatment outcomes are optimized.

## Figures and Tables

**Table 1:** Comparison of the ischemic stroke volume in 2020 and 2019 time periods.

Month	2020 *N* (%)	2019 *N* (%)	*P* value
Pre-COVID (Jan/Feb)	199 (45.2)	217 (36.4)	<0.001^∗^
Peak-COVID (Mar/Apr)	100 (22.7)	198 (33.2)	
Post-COVID (May/Jun)	141 (32.1)	181 (30.4)	
Total	440 (100%)	596 (100%)	

^∗^Statistical significance, *P* < 0.05.

**Table 2:** Demographic and Clinical Characteristics of the Study Cohort.

*N*	2020 Pre-COVID	2019 Pre-COVID	*P* value	2020 Pre-COVID	2019 Pre-COVID	*P* value	2020 Pre-COVID	2019 Pre-COVID	*P* value
	199	217		100	198		141	181	
	*N* (%)	*N* (%)		*N* (%)	*N* (%)		*N* (%)	*N* (%)	
*Age*									
18–39	0 (0.0)	4 (1.8)	*P* = 0.09	2 (2.0)	10 (5.1)	*P* = 0.56	4 (2.8)	4 (2.2)	*P* = 0.83
40–59	39 (19.6)	44 (20.3)		20 (20.0)	37 (18.7)		34 (24.1)	39 (21.6)	
60–79	88 (44.2)	108 (49.8)		46 (46.0)	82 (41.4)		57 (40.3)	82 (45.3)	
80+	72 (36.2)	61 (28.1)		32 (32.)	69 (34.9)		46 (32.6)	56 (30.9)	
*Sex*									
Female	106 (53.3)	99 (45.6)	*P* = 0.12	44 (44.0)	89 (45.0)	*P* = 0.88	69 (48.9)	88 (48.6)	*P* = 0.95
Male	93 (46.7)	118 (54.4)		56 (56.0)	109 (55.0)		72 (51.2)	93 (51.4)	
*NIHSSS*									
Unknown	28 (14.1)	32 (14.8)	*P* = 0.98	13 (13.0)	25 (12.6)	*P* = 0.99	16 (11.4)	26 (14.4)	*P* = 0.69
0-9	115 (57.8)	125 (57.6)		56 (56.0)	111 (56.1)		68 (48.2)	104 (57.5)	
10+	56 (28.1)	60 (27.7)		31 (31.0)	62 (31.3)		57 (40.4)	51 (28.2)	
*Mode of arrival*									
EMS	60 (30.2)	48 (22.1)	*P* = 0.09	34 (34)	61 (30.8)	*P* = 0.11	50 (35.5)	41 (22.7)	*P* = 0.09
Others/unknown	30 (15.1)	24 (11.1)		13 (13.0)	15 (7.6)		13 (9.2)	21 (11.6)	
Private	48 (24.1)	59 (27.2)		31 (31.0)	54 (27.3)		38 (27.0)	60 (33.2)	
Transfer	61 (30.7)	86 (39.6)		22 (22.0)	68 (34.3)		40 (28.4)	59 (32.6)	
*LKW to arrival*									
Unknown	40 (20.1)	58 (26.7)	*P* = 0.042^∗^	28 (28.0)	52 (26.3)	*P* = 0.03^∗^	35 (24.8)	39 (21.6)	*P* = 0.57
0–4.5	50 (25.1)	60 (27.7)		22 (22.0)	62 (31.3)		38 (27)	42 (23.2)	
4.5–6	8 (4.0)	6 (2.8)		8 (8.0)	7 (3.5)		6 (4.3)	8 (4.4)	
6–24	48 (24.1)	60 (27.7)		24 (24.0)	60 (30.3)		46 (32.6)	60 (33.2)	
>24	53 (26.6)	33 (15.2)		18 (18.0)	17 (8.6)		16 (11.3)	32 (17.7)	
*EVT*									
Not treated	183 (92.0)	199 (91.7)	*P* = 0.92	98 (98.0)	184 (92.9)	*P* = 0.07	125 (88.7)		*P* = 0.01^∗^
EVT	16 (8.0)	18 (8.3)		2 (2.0)	14 (7.1)		16 (11.3)	7 (3.9)	
*IVtPA*									
Not treated	160 (91.4)	211 (97.2)	*P* = 0.48	98 (98.0)	193 (97.5)	*P* = 0.78	130 (92.2)	171 (94.5)	*P* = 0.41
IVtPA	15 (8.6)	6 (2.8)		98 (98.0)	5 (2.5)		11 (7.8)	10 (5.5)	
*Discharge location*									
Expired	24 (12.1)	23 (10.6)	*P* = 0.85	12 (12.0)	20 (10.1)	*P* = 0.88	18 (12.8)	15 (8.3)	*P* = 0.34
Home	80 (40.2)	92 (42.4)		44 (44.0)	88 (44.4)		58 (41.1)	72 (39.8)	
Others	95 (47.7)	102 (47)		44 (44.0)	90 (45.5)		65 (46.1)	94 (51.9)	
*LOS*									
0–7	107 (61.1)	116 (53.5)	*P* = 0.65	109 (60.2)	121 (61.1)	*P* = 0.51			*P* = 0.34
8+	68 (38.9)	101 (46.5)		72 (39.8)	77 (38.9)		45 (31.9)	67 (37)	

NIHSS: National Institute of Health Stroke Scale; LKW: last known well; EMS: emergency medical services; EVT: endovascular thrombectomy; IVtPA: intravenous tissue plasminogen activator; LOS: length of stay. ^∗^*P* value < 0.05.

**Table 3:** Comparisons within 2020 time periods.

*N*	Pre-COVID	Peak-COVID	Post-COVID	*P* value
	199	100	141	
*Age*				
18–39	0 (0)	2 (2.0)	4 (2.8)	*P* = 0.31
40–59	39 (19.6)	20 (20.0)	24 (24.1)	
60–79	88 (44.4)	46 (46.0)	57 (40.4)	
80+	72 (36.2)	32 (32.0)	46 (32.6)	
*Sex*				
Female	106 (53.3)	44 (44.0)	69 (48.9)	*P* = 0.31
Male	93 (46.7)	56 (56.0	72 (51.1)	
*NIHSSS*				
Unknown	28 (14.1)	13 (13.0)	16 (11.4)	*P* = 0.21
0–9	115 (57.8)	56 (56.0)	68 (48.2)	
10+	56 (28.1)	31 (31.0)	57 (40.4)	
*Mode of arrival*				
EMS	60 (30.2)	34 (34.0)	50 (35.5)	*P* = 0.42
Others/Unknown	30 (15.1)	13 (13.0)	13 (9.2)	
Private	48 (24.1)	31 (31.0)	38 (27.0)	
Transfer	61 (30.6)	22 (22.0)	40 (28.3)	
*LKW to arrival*				
Unknown	40 (20.1)	28 (28.0)	35 (24.8)	*P* = 0.03^∗^
0–4.5	50 (25.1)	22 (22.0)	38 (27.0)	
4.5–6	8 (4.0)	8 (8.0)	6 (4.3)	
6–24	48 (24.1)	24 (24.0)	46 (32.6)	
>24	53 (26.6)	98 (98.0)	16 (11.4)	
*EVT*				
Not treated	183 (92.0)	98 (98.0)	125 (88.7)	*P* = 0.03^∗^
EVT	16 (8.0)	2 (2.0)	16 (11.4)	
*tPA*				
Not treated	191 (96.0)	98 (98.0)	130 (92.2)	*P* = 0.09
IVtPA	8 (4.0)	2 (2.0)	11 (7.8)	
*Discharge location*				
Expired	24 (12.1)	12 (12.0)	18 (12.8)	*P* = 0.98
Home	80 (40.2)	44 (44.0)	58 (41.1)	
Others	95 (47.7)	44 (44.0)	65 (46.1)	
*LOS*				
0–7	102 (51.3)	65 (65.0)	96 (68.1)	*P* < 0.01^∗^
8+	97 (48.7)	35 (35.0)	45 (31.9)	

NIHSS: National Institute of Health Stroke Scale; LKW: last known well; EMS: emergency medical services; EVT: endovascular therapy; IVtPA: intravenous tissue plasminogen activator; LOS: length of stay. ^∗^Statistical significance.

**Table 4:** Logistic regression models for in-hospital mortality during the control and 2020 Peak-COVID time periods.

Patient characteristics	Control period (January–June 2019)			Peak-COVID (March/April 2020)		
	Odds ratio	95% CI	*P* value	Odds ratio	95% CI	*P* value
Age ≥ 70 years	2.02	1.09–3.75	<0.0001	3.28	1.28–8.41	0.014
NIHSS ≥ 10	3.09	1.71–5.58	0.0002			
LOS ≥ 7 days	1.90	1.00–3.61	0.049	2.39	1.08–5.28	0.032
LKW ≥ 24 hours	4.50	1.47–13.80	0.009			

NIHSS: National Institute of Health Stroke Scale; LOS: length of stay; LKW: last known well.

## Data Availability

The data for this study is not publicly available.
